# Protein Tyrosine Phosphatase 1B Inhibitors from the Roots of *Cudrania tricuspidata*

**DOI:** 10.3390/molecules200611173

**Published:** 2015-06-17

**Authors:** Tran Hong Quang, Nguyen Thi Thanh Ngan, Chi-Su Yoon, Kwang-Ho Cho, Dae Gill Kang, Ho Sub Lee, Youn-Chul Kim, Hyuncheol Oh

**Affiliations:** 1 College of Pharmacy, Wonkwang University, Iksan 570-749, Korea; E-Mails: quangth2004@yahoo.com (T.H.Q.); nganthanh27@yahoo.com (N.T.H.N.); ycs1991@naver.com (C.-S.Y.); zxzxguild@gmail.com (K.-H.C.); 2 Institute of Marine Biochemistry, Vietnam Academy of Science and Technology (VAST), 18 Hoang Quoc Viet, Caugiay, Hanoi 10000, Vietnam; 3 Hanbang Body-Fluid Research Center, Wonkwang University, Iksan 570-749, Korea; E-Mails: dgkang.wku.ac.kr (D.G.K.); host@wku.ac.kr (H.S.L.); 4 Professional Graduate School of Oriental Medicine and College of Oriental Medicine, Wonkwang University, Iksan 570-749, Korea

**Keywords:** Moraceae, *Cudrania tricuspidata*, xanthones, flavonoids, PTP1B

## Abstract

A chemical investigation of the methanol extract from the roots of *Cudrania tricuspidata* resulted in the isolation of 16 compounds, including prenylated xanthones **1**–**9** and flavonoids **10**–**16**. Their structures were identified by NMR spectroscopy and mass spectrometry and comparisons with published data. Compounds **1**–**9** and **13**–**16** significantly inhibited PTP1B activity in a dose dependent manner, with IC_50_ values ranging from 1.9–13.6 μM. Prenylated xanthones showed stronger PTP1B inhibitory effects than the flavonoids, suggesting that they may be promising targets for the future discovery of novel PTP1B inhibitors. Furthermore, kinetic analyses indicated that compounds **1** and **13** inhibited PTP1B in a noncompetitive manner; therefore, they may be potential lead compounds in the development of anti-obesity and -diabetic agents.

## 1. Introduction

Protein tyrosine phosphatases (PTPs) constitute a large family of enzymes that are crucial modulators of tyrosine phosphorylation-dependent cellular events, such as growth, proliferation and differentiation, metabolism, immune response, cell-cell adhesion, and cell-matrix contacts [1,2]. The deregulation of PTP activity contributes to the pathogenesis of several human diseases, including cancer, diabetes, and immune disorders [2,3,4]. PTP1B, a member of the PTP superfamily, has emerged as the best-validated drug target for therapeutic development [5]. PTP1B is localized to the cytoplasmic face of the endoplasmic reticulum and is expressed ubiquitously, including in classically insulin-targeted tissues, such as liver, muscle, and fat [6]. PTP1B plays an important role in down-regulating insulin signaling cascades via tyrosine dephosphorylation of the insulin receptor, which renders it inactive, or dephosphorylation of insulin receptor substrates 1 and 2, which inhibits their interactions with downstream signaling molecules. PTP1B also negatively regulates the leptin signaling pathway by dephosphorylating JAK2, a phosphorylated tyrosine kinase, in the hypothalamus. This decreases food intake and increases energy expenditure [7,8,9,10]. *In vivo* studies have demonstrated an increase in insulin sensitivity, glycemic control, and resistance to a high fat diet in PTP1B-deficient mice [11,12]. Researches on PTP1B antisense oligonucleotides in diabetic animal models have also indicated that a reduction in PTP1B leads to a decrease in adipose tissue mass, plasma insulin, and blood glucose levels [13]. Quantitative trait loci and mutation analyses of the gene encoding PTP1B in humans have showed that the aberrant expression of PTP1B is involved in diabetes and obesity [14,15,16]. In addition, PTP1B was found to be overexpressed or up-regulated in human breast, colon, and ovarian cancers [17,18,19]. This biochemical, genetic, and pharmacological evidence suggests that the inhibition of PTP1B may be an effective strategy in the treatment of metabolic syndromes, such as type 2 diabetes and obesity and cancer.

*Cudrania tricuspidata* belongs to the Moraceae family and is widely distributed in Korea, Japan, and China. The roots of *C. tricuspidata* have been used in traditional medicine for the treatment of gonorrhea, rheumatism, jaundice, hepatitis, boils, scabies, bruising, and dysmenorrhea [20]. Previous studies have demonstrated that the major constituents of the roots of *C. tricuspidata* are xanthones [21,22,23] and flavonoids [24,25]. Biological effects of these components have been reported, including antioxidant [21], cytotoxic [22], brain monoamine oxidase (MAO) inhibition [23], anti-antherosclerotic and anti-inflammatory [26], and hepatoprotective [27] activities. In this paper, we describe the isolation and structural elucidation of 16 compounds, including nine prenylated xanthones and seven flavonoids, from the roots of *C. tricuspidata*. In addition, we evaluated of PTP1B inhibitory effects of these isolated compounds.

## 2. Results and Discussion

**Figure 1:**
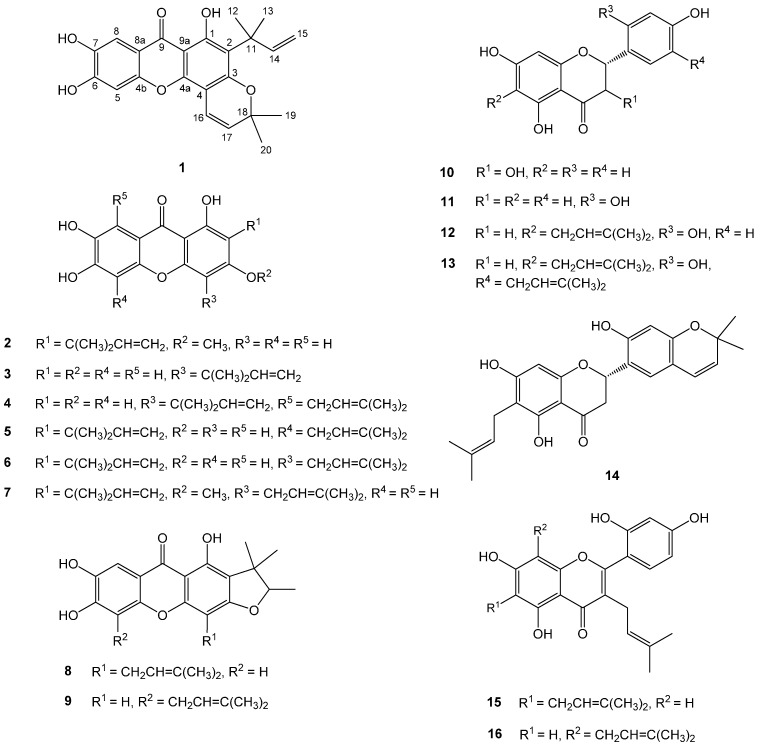
Chemical structures of compounds **1**–**16** from *Cudrania tricuspidata.*

Sixteen compounds were isolated from the roots of *C. tricuspidata* using various combined chromatographic methods. The NMR and MS data of the isolated compounds were analyzed and compared with those reported in the literature, allowing elucidation of the structures as cudratricusxanthone N (**1**) [28], 1,6,7-trihydroxy-2-(1,1-dimethyl-2-propenyl)-3-methoxyxanthone (**2**) [29], cudratricusxanthone L (**3**) [23], cudratricusxanthone A (**4**) [30], cudraxanthone L (**5**) [31], macluraxanthone B (**6**) [22], cudracuspixanthone A (**7**) [32], cudraxanthone D (**8**) [31], cudraxanthone M (**9**) [33], dihydrokaempferol (**10**) [34], steppogenin (**11**) [35], cudraflavanone B (**12**) [36], cudraflavanone D (**13**) [37], euchrestaflavanone C (**14**) [38], cudraflavone C (**15**) [39], and kuwanon C (**16**) [40], respectively (Figure 1).

PTP1B, a negative regulator in both insulin and leptin signaling, was evidenced as a promising drug target for type 2 diabetes and obesity. Its overexpression is involved in several human cancers, such as breast, colon, and ovarian cancers. Therefore, PTP1B inhibitors have emerged as attractive therapeutic targets for human disorders, such as diabetes, obesity, and cancer [41,42]. Research has led to the development of approximately 300 PTP1B inhibitors isolated from a variety of natural resources so far, of which two groups of phenolics (including flavonoids, bromophenols, phenolic acids, phenolics containing furan or pyran rings, coumarins, and lignans) and terpenoids (including sesquiterpenes, diterpenes, sesterterpenes, triterpenes, and steroids) have emerged as potential PTP1B inhibitors [43]. Several structure activity relationship (SAR) studies have determined some important chemical groups in the inhibition of PTP1B activity. For example, the preliminary SAR of flavonoids suggests that the presence of less polar substituents (such as an isoprenyl group), or conversion to less polar functionalities by methylation or acetylation of hydroxy group on the structures are usually beneficial to activity, while the addition of hydroxyl group may decrease activity [43]. These SAR results obtained could be helpful for designing drug candidates-like PTP1B inhibitor [43]. Although many natural PTP1B inhibitors showed promising clinical potential, there are no clinically used PTP1B inhibitors, which is most likely due to relatively low activities or lack of selectivity. Thus, searching for more potent and selective PTP1B inhibitors is still necessary.

The roots of *C. tricuspidata* and some isolated xanthones were shown to inhibit α-glucosidase activity [44,45]. In addition, *C. tricuspidata* roots induced hypoglycemia via lowering blood glucose in alloxan-induced hyperglycemic rats [46]. On the basis of these findings, we evaluated the inhibitory effects of the 16 isolated compounds on PTP1B activity. PTP1B enzyme (human, recombinant) was purchased from ATGen Co., Ltd. (Gyeonggi-do, Korea), and its activity was measured using *p*-nitrophenyl phosphate (*p*-NPP). The reaction mixture consisted of 50 mM Bis-Tris (pH 6.0), 2.0 mM EDTA, 5.0 mM dithiothreitol (DTT), PTP1B (0.04 μg), and 1.0 mM *p-*NPP, with or without test compounds. After incubation at 37.5 °C for 30 min, the reaction was stopped via the addition of 10 N NaOH. The production of *p*-nitrophenol (*p*-NP) was evaluated by measuring the absorbance at 405 nm. The non-enzymatic hydrolysis of 1.0 mM *p*-NPP was corrected by measuring the increase in absorbance at 405 nm in the absence of PTP1B enzyme [47]. As the result, we found that all tested compounds, except **10**–**12**, inhibited PTP1B activity in a dose dependent manner, with IC_50_ values ranging from 1.9–13.6 μM (Table 1).

**Table 1:** PTP1B inhibitory effects of compounds **1**–**16**.

Compounds	PTP1B Inhibitory Effects ^a^ (IC_50_ Values = μM)
**1**	2.0 ± 0.4
**2**	3.0 ± 0.6
**3**	3.0 ± 0.3
**4**	4.3 ± 0.6
**5**	4.6 ± 0.8
**6**	3.8 ± 0.5
**7**	1.9 ± 0.4
**8**	2.8 ± 0.6
**9**	3.5 ± 0.7
**10**	n.d.
**11**	n.d.
**12**	n.d.
**13**	5.7 ± 1.5
**14**	12.3 ± 2.2
**15**	9.4 ± 2.9
**16**	13.6 ± 3.3
Ursolic acid ^b^	3.8 ± 0.4

^a^ Values present mean ± SD of triplicate experiments; ^b^ Positive control; n.d.: not determined.

Previous studies have shown that many flavonoids can inhibit PTP1B, in particular the prenylated flavonoids and the prenyl group on ring B plays a key role in their inhibitory activities [43]. Accordingly, we found that, among the flavonoid analogues in our study, the strongest PTP1B inhibitory effect was from the prenylated flavonoid cudraflavanone D (**13**) (Table 1). Although flavonoids have received much attention in the development of anti-diabetic and -obesity drugs due to their potential to inhibit PTP1B, little is known about the PTP1B inhibitory effects of prenylated xanthones. As shown in Table 1, all prenylated xanthones **1**–**9** significantly inhibited PTP1B activity, with IC_50_ values ranging from 1.9–4.6 μM. A comparison of the IC_50_ values for the tested compounds indicated that the PTP1B inhibitory effects of the xanthones were stronger than those of the flavonoid derivatives (Table 1). This suggests that prenylated xanthones may be new therapeutic agents in the discovery and development of PTP1B inhibition-based anti-diabetic and -obesity drugs. To the best of our knowledge, this is the first report of the PTP1B inhibitory effects of the secondary metabolites isolated from *C. tricuspidata*. PTP1B plays an important role in the diabetes and obesity, therefore, *C. tricuspidata* root-induced hypoglycemia may be related to the PTP1B inhibitory effects of the isolated compounds reported in this study.

**Figure 2:**
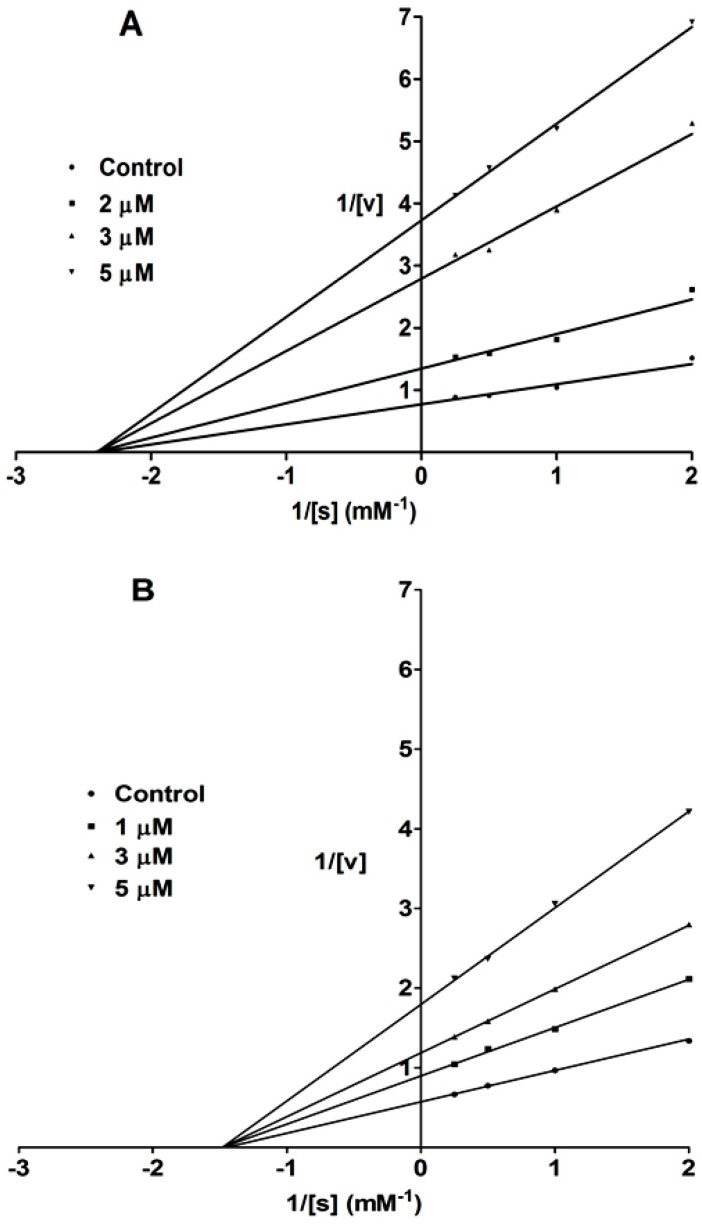
Lineweaver-Burk plots for inhibition of PTP1B-catalyzed hydrolysis of *p*-NPP by compounds **1** (**A**) and **13** (**B**). Data are expressed as mean initial velocity for triplicate experiments (*n* = 3) at each substrate concentration.

To elucidate the characteristics of PTP1B inhibition by the prenylated xanthones and flavonoids, compounds **1** and **13** were selected for an enzyme kinetic study. The kinetic studies were conducted using different concentrations of compounds **1**, **13**, and *p*-NPP. The initial rate was determined on the basis of the rate of increase in absorbance at 405 nm. The Michaelis–Menten constant (*K*_m_) and maximal velocity (*V*_max_) of PTP1B were determined by Lineweaver–Burk Plot analysis for competitive inhibition and the intercept on the vertical axis for noncompetitive inhibition. As shown in Figure 2, there was a reduction in *V*_max_ values, no change in *K*_m_ values, and the reciprocal plots of the tested compounds intersected to the left of the 1/V axis. This suggested that compounds **1** and **13** inhibited PTP1B activity in a noncompetitive pattern, with *K*_i_ values of 2.81 and 2.36, respectively. This implied that these compounds may bind to the enzyme-substrate complex or interact with an allosteric site which is distinct from the active site of the enzyme [48]. Recent studies have shown that it is difficult to identify a selective, safe, and effective PTP1B inhibitor. The major challenge is identifying inhibitors with pharmacologically acceptable bioavailability [49]. The PTP1B catalytic site contains the common structural motif of PTPs and is highly charged, while PTP1B inhibitors are positively charged; therefore, bioavailability is limited due to a low capacity to cross the plasma membrane [10]. This lack of cell permeability limits the utility of such compounds in signaling studies and further therapeutic development [49]. Recently, it has been suggested that noncompetitive PTP1B inhibitors that target the allosteric or active site of the enzyme in a distinct fashion could potentially develop into effective PTP1B inhibition-based drugs [48,49]. Accordingly, the two noncompetitive PTP1B inhibitors (**1** and **13**) found in the present study could be potential lead compounds for the development of anti-obesity and -diabetic drugs. Further studies to assess the selectivity, bioavailability, and efficacy of these compounds are necessary.

## 3. Experimental Section

### 3.1. General

NMR spectra (1D and 2D) were recorded using a JEOL JNM ECP-400 spectrometer (Tokyo, Japan) (400 MHz for ^1^H and 100 MHz for ^13^C). HMQC and HMBC experiments were optimized for ^1^*J*_CH_ = 140 Hz and ^n^*J*_CH_ = 8 Hz, respectively. ESIMS data were obtained using a Q-TOF micro LC-MS/MS instrument (Waters, Milford, MA, USA) at Korea University, Seoul, Korea. TLC was performed on Kieselgel 60 F_254_ (1.05715; Merck, Darmstadt, Germany) or RP-18 F_254s_ (Merck) plates. Spots were visualized by spraying with 10% aqueous H_2_SO_4_ solution, followed by heating. Column chromatography was performed on silica gel (Kieselgel 60, 70–230 mesh and 230–400 mesh, Merck) and YMC octadecyl-functionalized silica gel (C_18_).

### 3.2. Plant Material

The root barks of *Cudrania tricuspidata* were purchased in May 2014 at Daerim Korean crude drug store, Kumsan, Chungnam Province, Korea, and identified by Dr. Kyu-Kwan Jang, Botanical Garden, Wonkwang University. A voucher specimen (No. WP-2014-12) was deposited at the Herbarium of the College of Pharmacy, Wonkwang University (Iksan, Korea). 

### 3.3. Extraction and Isolation

Dried and pulverized roots of *C. tricuspidata* (6 kg) were extracted with MeOH (10 L) at room temperature. After concentration, the MeOH extract (300 g) was suspended in H_2_O (3 L) and partitioned successively with hexane (3 L) and CHCl_3_ (3 L) to give hexane (CTH), CHCl_3_ (CTC), and aqueous (CTW) fractions. The CTC fraction was chromatographed over a silica gel column, eluted with ethyl acetate (EtOAc) in hexane (20%–100%, step-wise), and washed with MeOH to provide six subfractions (CTC1-6). The CTC3 subfraction was separated by silica gel column chromatography (CC) and eluted with hexane-acetone (7:1–3:1, step-wise) to give four subfractions (CTC31-4). The CTC33 subfraction was subjected to a Sephadex LH-20 CC and eluted with CH_2_Cl_2_–MeOH (10:1) to provide four further subfractions (CTC331-4). Compounds **7** (50 mg) and **9** (45 mg) were isolated from subfraction CTC332 by a reversed phase (RP) C_18_ CC, using MeOH–H_2_O (7:1) as eluent. The CTC333 subfraction was separated by silica gel CC and eluted with CH_2_Cl_2_–EtOAc (30:1) to obtain **4** (20 mg), **5** (76 mg), and **14** (36 mg). The CTC4 subfraction was subjected to silica gel CC and eluted with CH_2_Cl_2_–MeOH (10:1) to provide four subfractions (CTC41-4). The CTC43 subfraction was separated by silica gel CC and eluted with hexane–acetone (5:1–1:1, stepwise) to give four subfractions (CTC431-4). Compounds **1** (27 mg), **2** (45 mg), **6** (55 mg), and **8** (5 mg) were isolated from subfraction CTC432 using silica gel CC and hexane–EtOAc (2:1) as eluent. The CTC433 subfraction was further separated by RP C_18_ CC and eluted with MeOH–H_2_O (7:1) to obtain **12** (72 mg), **15** (65 mg), and **16** (23 mg). The CTC43 subfraction was chromatographed over silica gel and eluted with hexane–acetone (5:1–1:1, stepwise) to give five subfractions (CTC431-5). The CTC442 subfraction was further purified by a RP C_18_ CC, using MeOH–H_2_O (3:1) as eluent, to provide **3** (6 mg) and **13** (60 mg). Compounds **10** (60 mg) and **11** (65 mg) were obtained from subfraction CTC443 by silica gel CC using hexane–EtOAc (3:1) as eluent.

### 3.4. PTP1B Inhibitory Activity Assay

PTP1B (human, recombinant) was purchased from ATGen Co., Ltd. (Gyeonggi-do, Korea). The enzyme activity was measured in a reaction mixture containing 1 mM *p-*NPP in 50 mM Bis-Tris (pH 6.0), 2.0 mM EDTA, and 5.0 mM dithiothreitol (DTT). The reaction mixture was placed in a 37.5 °C for 30 min, followed by termination of the reaction with the addition of 10 N NaOH. The amount of *p*-NP produced was estimated by measuring the increase in absorbance at 405 nm. The non-enzymatic hydrolysis of 1.0 mM *p-*NPP was corrected by measuring the increase in absorbance at 405 nm obtained in the absence of PTP1B enzyme. Inhibition kinetics studies were performed in the presence or absence of compounds **1** and **13** with different concentrations of *p*-NPP. Data were fitted by nonlinear regression analysis according to a Michaelis-Menten kinetic model using GraphPad Prism 5.01.

## 4. Conclusions 

Chemical study on the roots of *C. tricuspidata* resulted in the isolation of nine prenylated xanthones and seven flavonoids. Evaluation on the PTP1B inhibition showed that the inhibitory effects of the prenylated xanthones are stronger that those of flavonoids, suggesting that the prenylated xanthones could be considered as a new class for the discovery and development of the PTP1B inhibition based anti-diabetic and anti-obesity drugs. Further enzyme kinetic study indicated that compounds **1** and **13** inhibited PTP1B activity in a noncompetitive manner, suggesting that these compounds might be potential lead compounds for the development of anti-obesity and anti-diabetic drugs.

## Supplementary Data

Supplementary data, including HRMS and NMR spectra of compound **1**, ^1^H- and ^13^C-NMR data of compounds **1**–**16**, associated with this article can be accessed at: http://www.mdpi.com/1420-3049/20/06/11173/s1.
