# Supplementary material for: Protein Tyrosine Phosphatase 1B Inhibitors from the Roots of Cudrania tricuspidata
Source: Molecules. 2015 Jun 17;20(6):11173–83. doi: 10.3390/molecules200611173 (PMC6272669; doi:10.3390/molecules200611173)
Supplement: Supplementary file 1 [file molecules-20-11173-s001.pdf]

Supplementary Material

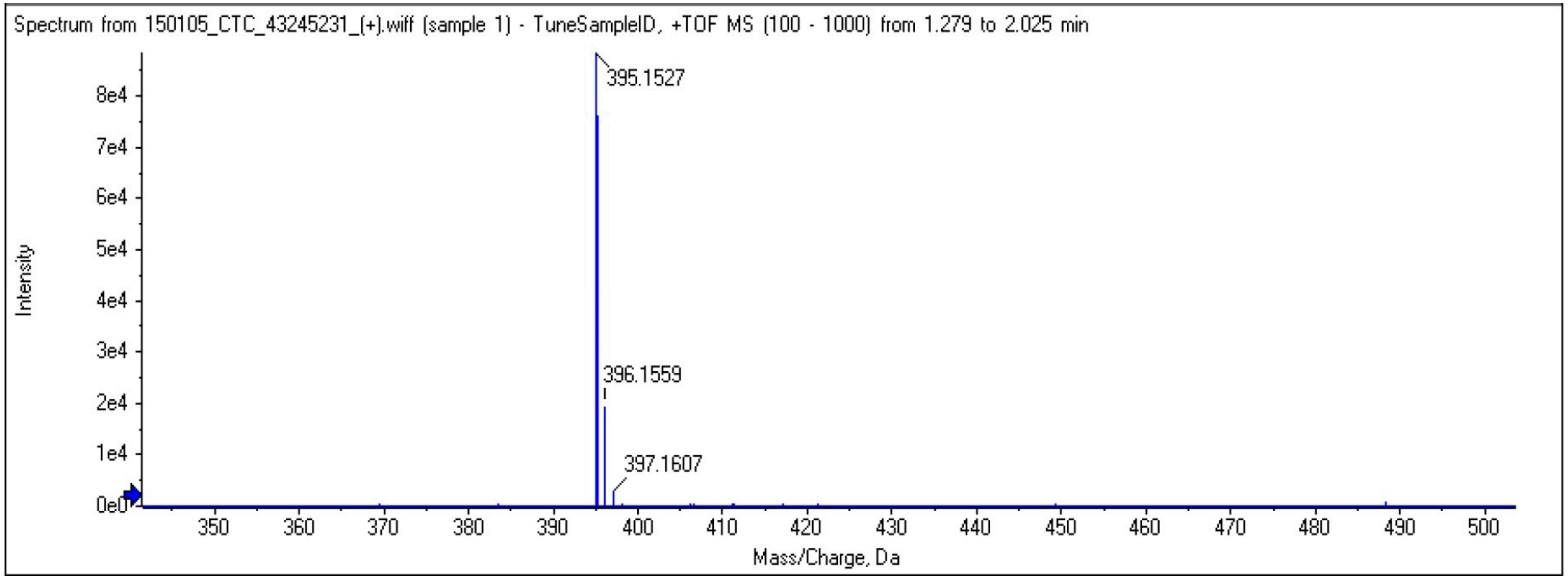

Figure S1. HRESITOF mass spectrum of **1**.

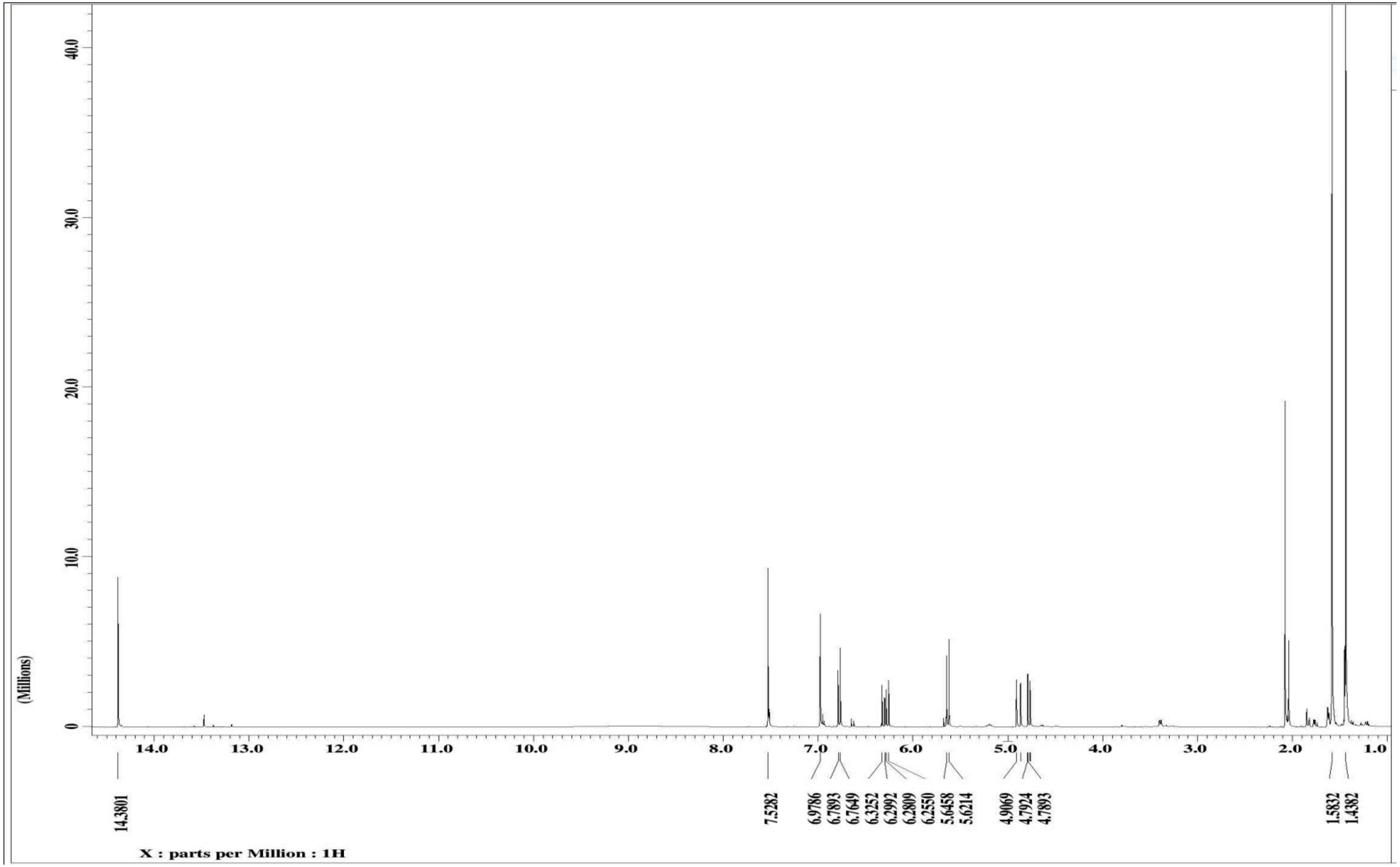

Figure S2. ^{1}H-NMR spectrum (400 MHz, Acetone-d_6) of compound **1**.

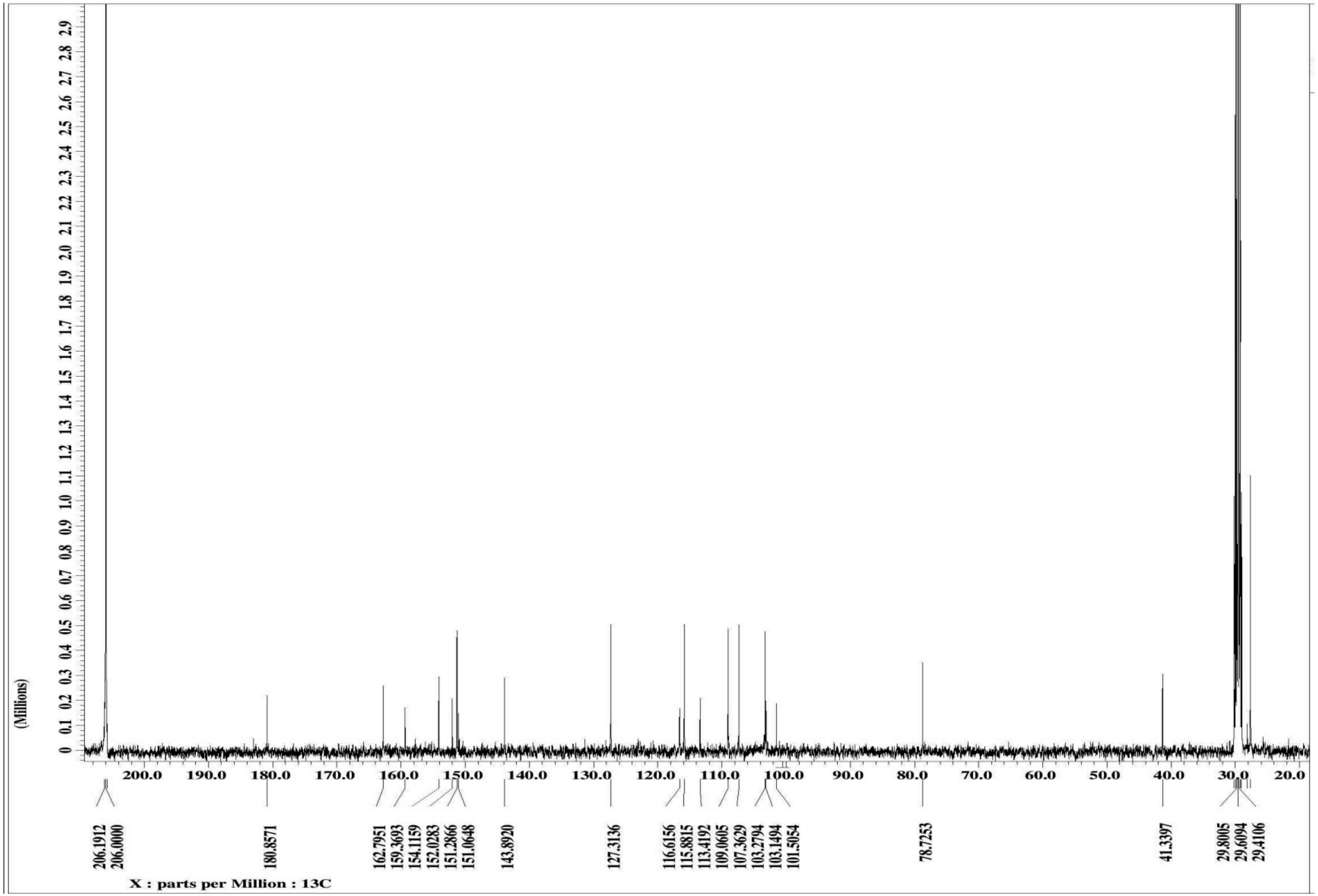

Figure S3. ^{13}C-NMR spectrum (100 MHz, Acetone-d_6) of compound **1**.

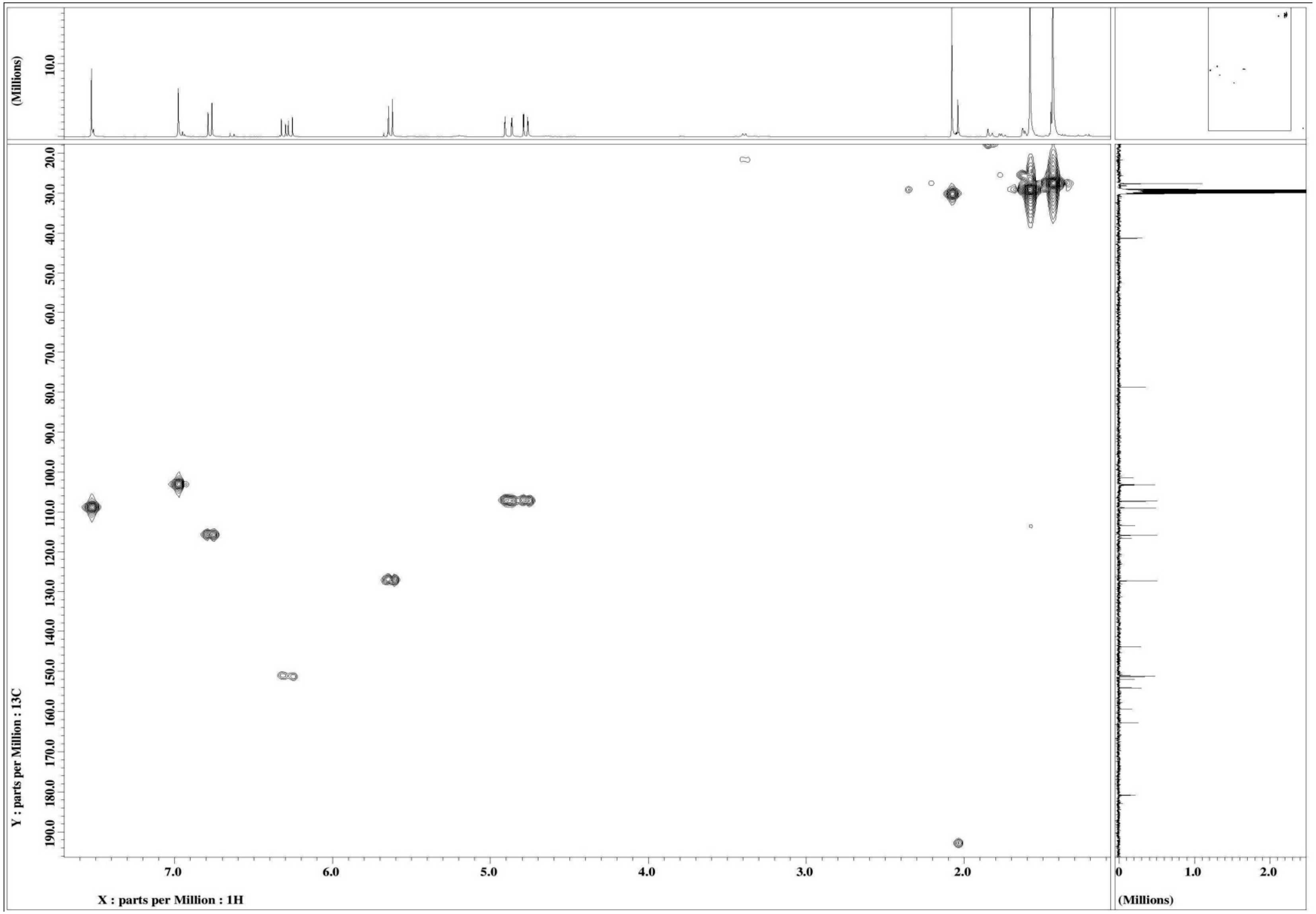

Figure S4. HMQC spectrum (400 MHz, Acetone-d_6) of compound **1**.

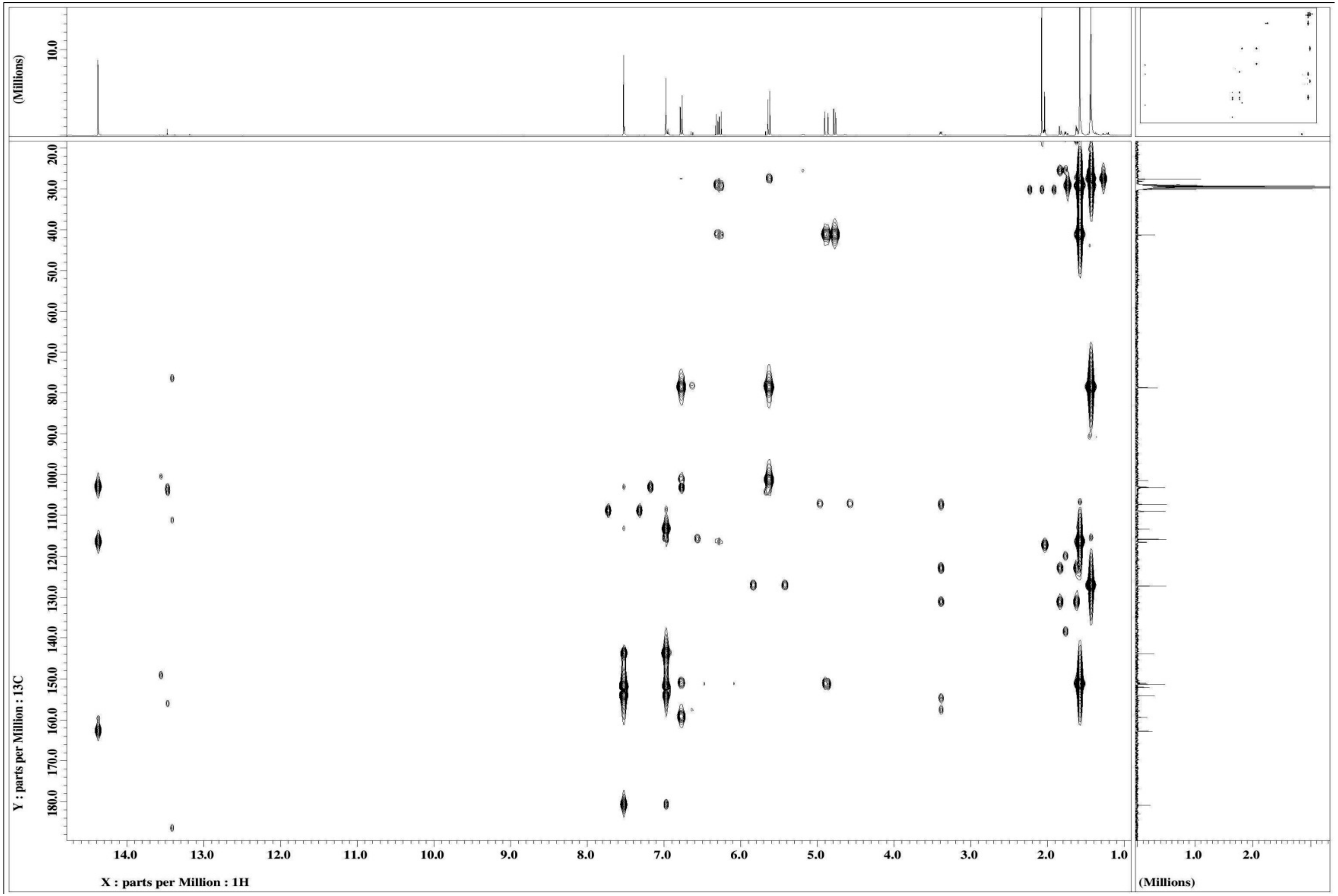

Figure S5. HMBC spectrum (400 MHz, Acetone-d_6) of compound **1**.

NMR Data of Compounds 1–16:

Cudratricusxanthone N (**1**): yellow gum. HRESIMS: *m/z* 395.1527 $[M + H]^+$ (calcd. for $C_{23}H_{23}O_6$, 395.1495). ^{1}H-NMR (Acetone-d_6, 400 MHz) δ 6.97 (s, H-5), 7.52 (s, H-8), 1.58 (s, H_3-12, H_3-13), 6.29 (dd, J = 10.4, 17.6 Hz, H-14), 4.90 (dd, J = 1.2, 17.6 Hz, H-15a), 4.76 (dd, J = 1.2, 10.8 Hz, H-15b), 6.78 (d, J = 10.0 Hz, H-16), 5.64 (d, J = 10.0 Hz, H-17), 1.43 (s, H_3-19, H_3-20), 14.38 (1-OH). ^{13}C-NMR (Acetone-d_6, 100 MHz) δ 162.7 (C-1), 116.6 (C-2), 159.3 (C-3), 101.5 (C-4), 151.0 (C-4a), 152.0 (C-4b), 103.2 (C-5), 154.1 (C-6), 143.8 (C-7), 109.0 (C-8), 113.4 (C-8a), 180.8 (C-9), 103.1 (C-9a), 41.3 (C-11), 29.2 (C-12, C-13), 151.2 (C-14), 107.3 (C-15), 115.8 (C-16), 127.3 (C-17), 78.7 (C-18), 27.6 (C-19, C-20).

1,6,7-Trihydroxy-2-(1,1-dimethyl-2-propenyl)-3-methoxyxanthone (**2**): pale yellow solid. ^{1}H-NMR (Acetone-d_6, 400 MHz) δ 6.33 (s, H-4), 6.79 (s, H-5), 7.40 (s, H-8), 1.57 (s, H_3-12, H_3-13), 6.25 (dd, J = 10.8, 17.6 Hz, H-14), 4.90 (dd, J = 1.2, 17.2 Hz, H-15a), 4.81 (dd, J = 1.2, 10.4 Hz, H-15b), 3.82 (s, 3-OCH_3). ^{13}C-NMR (Acetone-d_6, 100MHz) δ 160.5 (C-1), 111.5 (C-2), 163.8 (C-3), 93.7 (C-4), 152.7 (C-4a), 150.4 (C-4b), 100.9 (C-5), 153.9 (C-6), 142.2 (C-7), 106.6 (C-8), 111.0 (C-8a), 179.2 (C-9), 101.7 (C-9a), 39.8 (C-11), 27.7 (C-12, C-13), 149.8 (C-14), 105.1 (C-15), 53.9 (3-OCH_3).

Cudratricusxanthone L (**3**): pale yellow solid. ^{1}H-NMR (Acetone-d_6, 400 MHz) δ 6.21 (s, H-2), 6.91 (s, H-5), 7.50 (s, H-8), 1.66 (s, H_3-12, H_3-13), 6.36 (dd, J = 10.8, 17.2 Hz, H-14), 5.03 (d, J = 17.2 Hz, H-15a), 4.91 (d, J = 10.8 Hz, H-15b). ^{13}C-NMR (Acetone-d_6, 100 MHz) δ 161.9 (C-1), 99.3 (C-2), 163.7 (C-3), 111.4 (C-4), 156.6 (C-4a), 152.0 (C-4b), 102.9 (C-5), 153.9 (C-6), 143.7 (C-7), 108.6 (C-8), 113.0 (C-8a), 180.7 (C-9), 103.4 (C-9a), 41.5 (C-11), 29.3 (C-12, C-13), 151.2 (C-14), 107.7 (C-15).

Cudratricusxanthone A (**4**): yellow powder. ^{1}H-NMR (MeOD, 400 MHz) δ 6.10 (s, H-2), 6.66 (s, H-5), 1.61 (s, H_3-12, H_3-13), 6.30 (dd, J = 10.8, 17.2 Hz, H-14), 4.97 (dd, J = 1.2, 17.2 Hz, H-15a), 4.87 (dd, J = 1.6, 10.8 Hz, H-15b), 4.07 (d, J = 6.8 Hz, H-16), 5.24 (m, H-17), 1.63 (d, J = 1.2 Hz, H_3-19), 1.80 (s, H_3-20). ^{13}C-NMR (MeOD, 100MHz) δ 162.2 (C-1), 99.3 (C-2), 164.2 (C-3), 111.4 (C-4), 156.3 (C-4a), 153.2 (C-4b), 100.5 (C-5), 153.7 (C-6), 142.0 (C-7), 129.0 (C-8), 111.6 (C-8a), 183.9 (C-9), 104.5 (C-9a), 41.9 (C-11), 29.6 (C-12, C-13), 152.1 (C-14), 107.5 (C-15), 26.5 (C-16), 124.7 (C-17), 131.7 (C-18), 26.0 (C-19), 18.2 (C-20).

Cudraxanthone L (**5**): yellow powder. ^{1}H-NMR (MeOD, 400 MHz) δ 6.22 (s, H-4), 7.26 (s, H-8), 1.56 (s, H_3-12, H_3-13), 6.32 (dd, J = 10.0, 17.6 Hz, H-14), 4.90 (dd, J = 1.2, 17.6 Hz, H-15a), 4.80 (dd, J = 1.2, 10.4 Hz, H-15b), 3.45 (d, J = 7.2 Hz, H-16), 5.18 (m, H-17), 1.60 (s, H_3-19), 1.81 (s, H_3-20). ^{13}C-NMR (MeOD, 100 MHz) δ 163.1 (C-1), 116.4 (C-2), 165.0 (C-3), 94.9 (C-4), 156.9 (C-4a), 150.6 (C-4b), 115.6 (C-5), 152.1 (C-6), 143.4 (C-7), 106.0 (C-8), 113.3 (C-8a), 180.4 (C-9), 103.1 (C-9a), 41.9 (C-11), 29.2 (C-12, C-13), 151.4 (C-14), 108.1 (C-15), 23.1 (C-16), 122.6 (C-17), 132.4 (C-18), 25.8 (C-19), 18.0 (C-20).

Macluraxanthone B (**6**): pale yellow solid. ^{1}H-NMR (Acetone-d_6, 400 MHz) δ 6.92 (s, H-5), 7.52 (s, H-8), 1.61 (s, H_3-12, H_3-13, H_3-20), 6.49 (dd, J = 10.4, 18.0 Hz, H-14), 5.44 (dd, J = 1.2, 18.0 Hz, H-15a), 5.33 (dd, J = 1.2, 10.4 Hz, H-15b), 3.40 (d, J = 6.8 Hz, H-16), 5.17 (m, H-17), 1.82 (s, H_3-19).

^{13}C-NMR (Acetone-d_6, 100 MHz) δ 160.5 (C-1), 113.4 (C-2), 160.9 (C-3), 106.9 (C-4), 153.7 (C-4a), 151.9 (C-4b), 102.9 (C-5), 153.5 (C-6), 143.4 (C-7), 108.8 (C-8), 113.0 (C-8a), 180.7 (C-9), 102.9 (C-9a), 41.5 (C-11), 27.4 (C-12, C-13), 150.1 (C-14), 112.7 (C-15), 22.0 (C-16), 122.9 (C-17), 131.2 (C-18), 17.6 (C-19), 25.4 (C-20).

Cudracuspixanthone A (**7**): pale yellow solid. ^{1}H-NMR (Acetone-d_6, 400 MHz) δ 6.92 (s, H-5), 7.53 (s, H-8), 1.57 (s, H_3-12, H_3-13), 6.43 (dd, J = 10.8, 17.2 Hz, H-14), 4.92 (dd, J = 1.2, 17.2 Hz, H-15a), 4.73 (dd, J = 1.2, 10.8 Hz, H-15b), 3.44 (d, J = 7.2 Hz, H-16), 5.21 (m, H-17), 1.83 (s, H_3-19), 1.64 (s, H_3-20), 3.54 (s, 3-OCH_3). ^{13}C-NMR (Acetone-d_6, 100 MHz) δ 161.0 (C-1), 122.5 (C-2), 164.0 (C-3), 113.6 (C-4), 154.2 (C-4a), 152.2 (C-4b), 103.0 (C-5), 153.7 (C-6), 143.7 (C-7), 108.8 (C-8), 113.1 (C-8a), 181.3 (C-9), 105.6 (C-9a), 41.2 (C-11), 27.1 (C-12, C-13), 150.3 (C-14), 104.3 (C-15), 23.0 (C-16), 123.5 (C-17), 131.4 (C-18), 17.7 (C-19), 25.4 (C-20), 62.5 (3-OCH_3).

Cudraxanthone D (**8**): pale yellow solid. ^{1}H-NMR (Acetone-d_6, 400 MHz) δ 6.96 (s, H-5), 7.53 (s, H-8), 1.47 (s, H_3-12), 1.22 (s, H_3-13), 4.52 (d, J = 6.8 Hz, H-14), 1.39 (d, J = 6.8 Hz, H-15), 3.40 (d, J = 7.2 Hz, H-16), 5.25 (m, H-17), 1.64 (s, H_3-19), 1.84 (s, H_3-20). ^{13}C-NMR (Acetone-d_6, 100MHz) δ 157.1 (C-1), 116.4 (C-2), 164.0 (C-3), 102.6 (C-4), 155.5 (C-4a), 152.4 (C-4b), 103.3 (C-5), 153.9 (C-6), 143.8 (C-7), 109.0 (C-8), 113.4 (C-8a), 180.9 (C-9), 104.0 (C-9a), 44.2 (C-11), 20.8 (C-12), 25.4 (C-13), 91.1 (C-14), 14.5 (C-15), 22.4 (C-16), 122.7 (C-17), 131.9 (C-18), 17.8 (C-19), 25.7 (C-20).

Cudraxanthone M (**9**): white amorphous powder. ^{1}H-NMR (Acetone-d_6, 400 MHz) δ 6.31 (s, H-4), 7.45 (s, H-8), 1.22 (s, H_3-12), 1.46 (s, H_3-13), 4.48 (d, J = 6.8 Hz, H-14), 1.37 (d, J = 6.4 Hz, H_3-15), 3.60 (d, J = 7.2 Hz, H-16), 5.37 (m, H-17), 1.64 (s, H_3-19), 1.87 (s, H_3-20). ^{13}C-NMR (Acetone-d_6, 100 MHz) δ 158.8 (C-1), 117.0 (C-2), 166.1 (C-3), 89.7 (C-4), 158.6 (C-4a), 150.6 (C-4b), 116.3 (C-5), 151.8 (C-6), 143.2 (C-7), 106.1 (C-8), 113.2 (C-8a), 181.0 (C-9), 103.9 (C-9a), 43.9 (C-11), 25.4 (C-12), 20.8 (C-13), 91.49 (C-1), 14.5 (C-15), 23.0 (C-16), 122.4 (C-17), 132.3 (C-18), 25.8 (C-19), 18.0 (C-20).

Dihydrokaempferol (**10**): White solid. ^{1}H-NMR (DMSO-d_6, 400 MHz) δ 5.07 (d, J = 11.2 Hz, H-2), 4.60 (d, J = 11.6 Hz, H-3), 5.89 (d, J = 2.0 Hz, H-6), 5.95 (d, J = 2.0 Hz, H-8), 7.34 (d, J = 8.4 Hz, H-2′, H-6′), 6.83 (d, J = 8.4 Hz, H-3′, H-5′). ^{13}C-NMR (DMSO-d_6, 100 MHz) δ 82.8 (C-2), 71.4 (C-3), 197.9 (C-4), 163.3 (C-5), 96.0 (C-6), 166.7 (C-7), 95.0 (C-8), 162.5 (C-9), 100.4 (C-10), 127.5 (C-1′), 129.4 (C-2′, 6′), 114.9 (C-3′, 5′), 157.7 (C-4′).

Steppogenin (**11**): brown solid. ^{1}H-NMR (DMSO-d_6, 400 MHz) δ 5.61 (dd, J = 2.8, 13.2 Hz, H-2), 3.25 (dd, J = 13.2, 17.2 Hz, H-3a), 2.62 (dd, J = 2.8, 17.2 Hz, H-3b), 5.89 (br s, H-6, H-8), 6.37 (d, J = 1.6 Hz, H-3′), 6.29 (d, J = 1.6, 8.4 Hz, H-5′), 7.20 (d, J = 8.4 Hz, H-6′). ^{13}C-NMR (DMSO-d_6, 100 MHz) δ 74.0 (C-2), 41.3 (C-3), 197.0 (C-4), 163.7 (C-5), 95.9 (C-6), 166.7 (C-7), 95.1 (C-8), 163.6 (C-9), 101.9 (C-10), 115.6 (C-1′), 155.9 (C-2′), 102.6 (C-3′), 158.8 (C-4′), 106.7 (C-5′), 128.4 (C-6′).

Cudraflavanone B (**12**): brown yellow solid. ^{1}H-NMR (Acetone-d_6, 400 MHz) δ 5.69 (dd, J = 3.2, 12.8 Hz, H-2), 3.15 (dd, J = 12.8, 17.2 Hz, H-3a), 2.72 (dd, J = 3.2, 17.2 Hz, H-3b), 6.03 (s, H-8), 6.46 (s, H-3′), 6.43 (dd, J = 2.4, 8.0 Hz, H-5′), 7.30 (d, J = 8.0 Hz, H-6′), 3.25 (d, J = 7.2 Hz, H-1″), 5.23 (m, H-2″), 1.64 (s, H_3-4″), 1.73 (s, H_3-5″), 12.47 (s, 5-OH). ^{13}C-NMR (Acetone-d_6, 100MHz) δ 74.3 (C-2),

41.7 (C-3), 196.9 (C-4), 161.5 (C-5), 108.0 (C-6), 163.7 (C-7), 94.3 (C-8), 161.3 (C-9), 102.1 (C-10), 116.6 (C-1′), 155.3 (C-2′), 102.6 (C-3′), 158.5 (C-4′), 107.0 (C-5′), 128.0 (C-6′), 20.7 (C-1″), 122.7 (C-2″), 130.30 (C-3″), 24.97 (C-4″), 16.95 (C-5″).

Cudraflavanone D (**13**): brown oil. ^{1}H-NMR (MeOD, 400 MHz) δ 5.58 (dd, *J* = 3.2, 12.4 Hz, H-2), 3.03 (dd, *J* = 12.0, 17.2 Hz, H-3a), 2.71 (dd, *J* = 3.2, 17.2 Hz, H-3b), 5.93 (s, H-8), 6.32 (s, H-3'), 7.03 (s, H-6′), 3.18 (d, *J* = 7.2 Hz, H-11), 5.18 (m, H-12), 1.73 (s, H_3-14), 1.65 (s, H_3-15), 3.18 (d, *J* = 7.2 Hz, H-16), 5.25 (m, H-17), 1.63 (s, H_3-19), 1.69 (s, H_3-20). ^{13}C-NMR (MeOD, 100 MHz) δ 75.9 (C-2), 43.1 (C-3), 198.4 (C-4), 162.4 (C-5), 109.4 (C-6), 165.8 (C-7), 95.3 (C-8), 162.9 (C-9), 103.2 (C-10), 117.5 (C-1′), 154.3 (C-2′), 103.3 (C-3′), 156.8 (C-4′), 120.5 (C-5′), 128.6 (C-6′), 21.8 (C-11), 123.9 (C-12), 131.5 (C-13), 17.8 (C-14), 25.9 (C-15), 28.6 (C-16), 124.3 (C-17), 132.5 (C-18), 25.9 (C-19), 17.7 (C-20).

Euchrestaflavanone C (**14**): brown solid. ^{1}H-NMR ($CDCl_3$, 400 MHz) δ 5.56 (dd, *J* = 2.8, 12.8 Hz, H-2), 3.05 (dd, *J* = 12.8, 17.2 Hz, H-3a), 2.85 (dd, *J* = 3.2, 17.2 Hz, H-3b), 5.97 (s, H-8), 6.29 (s, H-3′), 6.94 (s, H-6′), 3.29 (d, *J* = 7.2 Hz, H-11), 5.21 (m, H-12), 1.69 (s, H_3-14), 1.77 (s, H_3-15), 6.24 (d, *J* = 10.0 Hz, H-16), 5.46 (d, *J* = 10.0 Hz, H-17), 1.38 (s, H_3-20, H_3-21). ^{13}C-NMR ($CDCl_3$, 100 MHz) δ 75.4 (C-2), 41.7 (C-3), 197.0 (C-4), 161.3 (C-5), 108.0 (C-6), 163.9 (C-7), 95.3 (C-8), 160.9 (C-9), 102.7 (C-10), 117.0 (C-1′), 154.4 (C-2′), 104.2 (C-3′), 154.1 (C-4′), 114.4 (C-5′), 124.6 (C-6′), 21.0 (C-11), 121.6 (C-12), 133.8 (C-13), 25.7 (C-14), 17.7 (C-15), 121.6 (C-16), 128.1 (C-17), 76.6 (C-18), 27.9 (C-20, C-21).

Cudraflavone C (**15**): brown yellow oil. ^{1}H-NMR (Acetone-d_6, 400 MHz) δ 6.37 (s, H-8), 6.54 (d, *J* = 2.4 Hz, H-3′), 6.50 (dd, *J* = 2.4, 8.4 Hz, H-5′), 7.17 (d, *J* = 8.4 Hz, H-6′), 3.35 (d, *J* = 7.2 Hz, H-11), 5.26 (m, H-12), 1.41 (s, H_3-14), 1.62 (s, H_3-15), 3.10 (d, *J* = 6.8 Hz, H-16), 5.11 (d, *J* = 6.8 Hz, H-17), 1.55 (s, H_3-19), 1.76 (s, H_3-20). ^{13}C-NMR (Acetone-d_6, 100MHz) δ 161.2 (C-2), 120.8 (C-3), 182.2 (C-4), 159.3 (C-5), 111.0 (C-6), 161.5 (C-7), 92.8 (C-8), 156.3 (C-9), 104.3 (C-10), 112.3 (C-1′), 156.2 (C-2′), 103.1 (C-3′), 160.5 (C-4′), 107.3 (C-5′), 131.5 (C-6'), 23.9 (C-11), 122.6 (C-12), 131.2 (C-13), 25.1 (C-14), 17.2 (C-15), 21.2 (C-16), 121.9 (C-17), 130.7 (C-18), 25.1 (C-19), 16.9 (C-20).

Kuwanon C (**16**): brown oil. ^{1}H-NMR (Acetone-d_6, 400 MHz) δ 6.31 (s, H-6), 6.56 (d, *J* = 2.4 Hz, H-3′, 6.52 (dd, *J* = 2.4, 8.4 Hz, H-5′), 7.21 (d, *J* = 8.4 Hz, H-6′), 3.12 (d, *J* = 7.2 Hz, H-11), 5.12 (m, H-12), 1.55 (s, H_3-14), 1.41 (s, H_3-15), 3.35 (d, *J* = 7.2 Hz, H-16), 5.19 (m, H-17), 1.56 (s, H_3-19, H_3-20). ^{13}C-NMR (Acetone-d_6, 100 MHz) δ 161.6 (C-2), 120.5 (C-3), 182.7 (C-4), 160.1 (C-5), 98.1 (C-6), 161.1 (C-7), 106.0 (C-8), 156.6 (C-9), 104.5 (C-10), 112.4 (C-1′), 155.8 (C-2′), 103.1 (C-3′), 160.7 (C-4′), 107.3 (C-5′), 131.6 (C-6″), 23.9 (C-11), 122.4 (C-12), 131.3 (C-13), 17.0 (C-14), 25.2 (C-15), 21.4 (C-16), 122.1 (C-17), 130.9 (C-18), 25.1 (C-19), 17.0 (C-20).
